# Case Report: Identification of rare *H3-3A* p.G35W variant in a case of adolescent tectal plate low-grade glioma

**DOI:** 10.3389/fonc.2026.1772874

**Published:** 2026-04-30

**Authors:** Andy Ung, Vanesa M. Tomatis, Esther Quick, Laveniya Satgunaseelan, Ema Knight, Chrisovalantis Tsimiklis, Elyse C Page, Jordan R. Hansford, Annika R. Mascarenhas

**Affiliations:** 1 Department of Surgical and Perioperative Medicine, Flinders Medical Centre, Bedford Park, SA, Australia; 2 Department of Neurosurgery, Flinders Medical Centre, Bedford Park, SA, Australia; 3 Department of Surgical Pathology, Flinders Medical Centre, Bedford Park, SA, Australia; 4 Department of Neuropathology, Royal Prince Alfred Hospital, Camperdown, NSW, Australia; 5 South Australian Immunogenomics Cancer Institute, Faculty of Health and Medical Sciences, The University of Adelaide, Adelaide, SA, Australia; 6 Precision Cancer Medicine Theme, South Australian Health and Medical Research Institute, Adelaide, SA, Australia; 7 Michael Rice Centre for Haematology and Oncology, Women’s and Children’s Hospital, North Adelaide, SA, Australia

**Keywords:** G34W-mutant, methylation profiling, paediatric, pilocytic astrocytoma, tectal plate glioma

## Abstract

The treatment of paediatric low-grade gliomas has been previously controversial as to whether upfront surgery or a conservative approach should be utilised, with a recent paradigm shift favouring biopsy or resection, if possible, and early treatment. Pilocytic astrocytomas are a subset of low-grade glioma with favourable prognoses. Here, we present the case of an adolescent who underwent a stereotactic endoscopic biopsy of a tectal plate lesion, classified as a pilocytic astrocytoma. Due to the intricate nature of this tumour, methylation and targeted sequencing were performed, which identified a rare *H3-3A* p.G35W(G34W) variant. This variant has been predominantly identified in giant cell tumours of the bone, with isolated reports in spinal cord astrocytomas. In the current WHO Classification of Tumours of the Central Nervous System, diffuse hemispheric glioma, H3 G34-mutant is recognised as a distinct tumour type with frequent *TP53*, *PDGFRA*, and-ATRX and DAXX alterations and is associated with aggressive behaviour. The histone H3 variant (*H3-3A* p.G35W) detected in our patient has not been previously characterised in tectal plate gliomas, rendering it an unclear predictor of tumour behaviour. In this case study, we discuss the importance of methylation profiling and the potential implications of H3 G34-mutant gliomas.

## Introduction

Tectal plate gliomas are a rare paediatric predominant midline tumour often histologically reported to be low-grade pilocytic astrocytoma ([PA] WHO grade 1) or diffuse astrocytoma ([DA] WHO grade 2), contributing to less than 5% of brainstem tumours diagnosed in children (1, 2). Molecular diagnostics of low-grade astrocytoma is not frequently performed as most cases in the recent past have not progressed to biopsy and have been managed based on radiographic disease progression and symptomatology. Paediatric gliomas have recently been uniquely defined in the 2021 WHO (3), recognising the unique molecular features in both low- and high-grade gliomas. Low-grade glioma (LGG) typically consists of tumours with pathogenic alterations in signalling through the MAP-kinase pathway, predominantly with changes in *BRAF* (4). Our understanding of paediatric and adolescent high-grade glioma (HGG), however, consists of developmentally stalled tumours with specific changes in epigenetic mediators, predominantly including histone-mediated changes in hemispheric and diffuse midline glioma (DMG) (5–7). Recently, the cell of origin in both DMG (oligodendroglial precursor cells [OPC]) (8) and hemispheric HGG (interneurons) (9) have been discovered, highlighting these stalled developmental pathways. In an integrated analysis of more than 1000 paediatric HGG and DMG cases, recurrent mutations involving genes encoding histones H3.3 and H3.1, with H3 K27-altered, H3 G34-mutant, and H3-wildtype variants being distinct subgroups of paediatric diffuse HGG (10).

Histone proteins regulate and mediate DNA (deoxyribonucleic acid) organisation, playing a crucial role in the epigenetic regulation of gene expression (11). Each nucleosome, the basic structural unit of DNA, comprises DNA and two copies of each core histone: H2A, H2B, H3 and H4 (11). Compared to canonical histone H3 isoforms (H3.1 and H3.2), the non-canonical H3.3 isoform is the most mutated histone protein reported in human cancer (12). Amongst the most documented examples of histone H3.3 mutants are H3.3 p.G35W(G34W) mostly found in giant cell tumour of bone (GCTB), H3.3 p.K27M(K36M) in chondroblastoma, as well as H3.3 p.G35R/V(G34R/V) and H3.3 p.K28M(K27M) in paediatric-type diffuse HGG (13, 14). Point mutations in the *H3-3A* gene, encoding oncohistone H3.3, have been fundamental to elucidating the molecular pathogenesis of paediatric gliomas.

The natural history of tectal plate gliomas is indolent with slow growth. A retrospective analysis comprising 170 patients from a single institute demonstrated that 86% of patients had radiographic progression-free survival at a ten-year follow-up from diagnosis (median age of 24 years, age range 0–73 years, 67 patients were less than 18 years of age) (15). Due to the anatomical approximation of the tectum mesencephali to the aqueduct of Sylvius, patients tend to clinically present with signs and symptoms of raised intracranial pressure secondary to obstructive hydrocephalus (16). Although less frequently reported, patients can present with Parinaud’s syndrome (dorsal midbrain syndrome or pretectal syndrome), a clinical triad of supranuclear vertical conjugate gaze paralysis, convergence retraction nystagmus, and dissociated pupillary response to light due to the compression of decussating posterior commissure fibres between the pineal gland and the superior colliculus (17).

Management of obstructive hydrocephalus through cerebral spinal fluid (CSF) diversion procedures remains the cornerstone of therapy as a sequela of the tumour rather than targeted therapy of the tumour itself (18). Paediatric LGG have a favourable overall survival (OS), with adult survivors of paediatric disease shown to have a low incidence of glioma-related death, with reports of greater than 90% survival at 20–25 years after diagnosis in the absence of aggressive treatment modalities such as radiotherapy (19, 20).

In our report, we describe for the first time the finding of likely oncogenic histone H3.3 variant, *H3-3A* p.G35W (glycine to tryptophan missense mutation) in a case of adolescent tectal plate low-grade glioma. Following this finding and the limited understanding of the role of this mutation in tumour progression and behaviour, we discuss the importance of further molecular studies, including methylation profiling, to aid therapeutic decision making and close surveillance of the patient and tumour evolution.

## Case presentation

**Figure 1:**
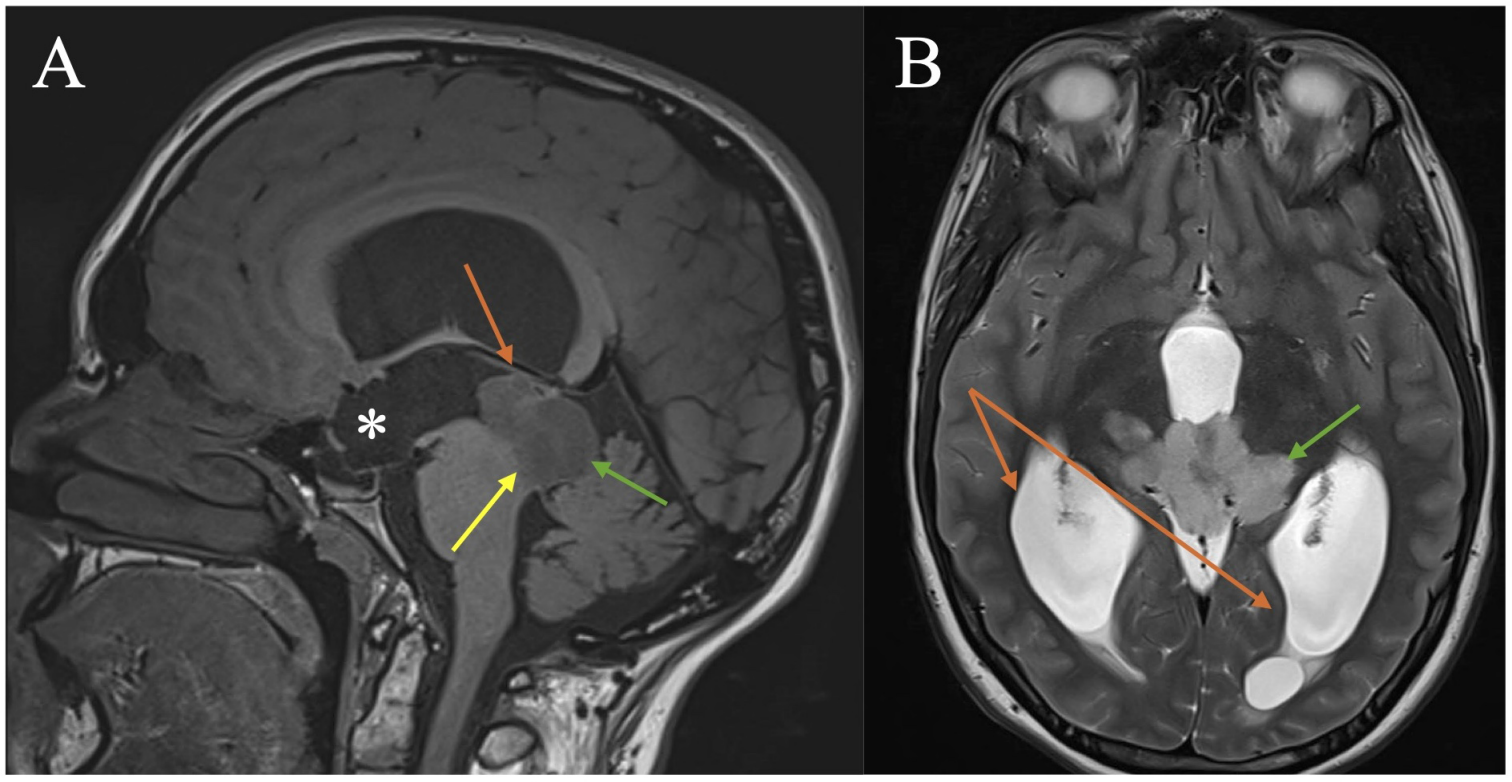
lndex MRI brain depicting tectal plate glioma identified in a 17-year-old female patient. **(A)** Sagittal MRI T1-weighted image demonstrating a hypointense pineal region mass (green arrow) displacing the pineal gland (orange arrow) superiorly. There is dilatation of the third ventricle with bowing anteriorly of the anterior wall of the third ventricle (lamina terminalis) and displacement of the floor of the third ventricle inferiorly (*). Severe narrowing of the cerebral aqueduct is observed (yellow arrow). **(B)** Axial MRI T2-weighted image demonstrating a hyperintense lesion centred on the tectum with infiltration into the thalami, right more than left (green arrow). Dilated posterior horn of lateral ventricles at level of pineal region lesion (orange arrow).

The patient is a left-hand dominant 17-year-old girl with no prior medical co-morbidities; she was referred to the emergency department via her local optometrist due to findings of bilateral optic disc swelling. She initially presented with progressive visual disturbances over a month. She also reported a new pattern of non-positional bi-occipital morning headaches occurring 1–2 times weekly, which self-resolved over the last two months. Additionally, a new onset of left-sided facial weakness and numbness with difficulty closing her left eye for the preceding week was revealed. The patient had initial concerns about updating her prescription glasses and presented to her optometrist with a prior ocular history of phoria requiring multifocal lenses. On examination, her aided visual acuity was 6/9.5 in the right eye and 6/15 in the left eye. Air puff tonometry revealed intraocular pressures of 17 mmHg in the right eye and 19 mmHg in the left eye; Ishihara test was 10/13 bilaterally, and ocular motility demonstrated restriction of the lateral rectus with diplopia. Slit lamp microscopy revealed pink discs with grossly raised margins in both eyes with no other reported abnormalities. On neurological examination, the patient had a House-Brackmann grade 3 left-sided cranial nerve seven palsy. MRI revealed a non-contrast enhancing, T2 hyperintense, and T1 hypointense lesion of 49x28x31 mm involving the tectal plate with displacement of the pineal gland superiorly, with infiltration into the thalami and right crus cerebri (**Figures 1A, B**). Most tectal lesions are usually confined to the tectal plate. MRI also revealed complete obstruction of the cerebral aqueduct and associated triventricular hydrocephalus with transependymal oedema. No leptomeningeal enhancement or diffusion restriction was observed. Drop metastases were not observed with subsequent MRI of the spine. The patient progressed to CSF diversion and tissue sampling as a priority.

A right frontal approach, endoscopic third ventriculostomy, and endoscopic biopsy of the tectal plate lesion were performed to guide further treatment based on histology and molecular studies. MRI CSF flow studies were performed post ventriculostomy, demonstrating a patent stoma. From the patient’s perspective, diagnostic challenges included timing of interventional and diagnostic tests, alongside the patients’ schooling commitments. The patient’s presenting symptoms resolved post-operation and the patient resumed normal activities of daily living, however, fundoscopy still demonstrated optic disc swelling. The patient was subsequently discharged from her index admission pending further biopsy analyses (**Supplementary Figure 1**).

**Figure 2:**
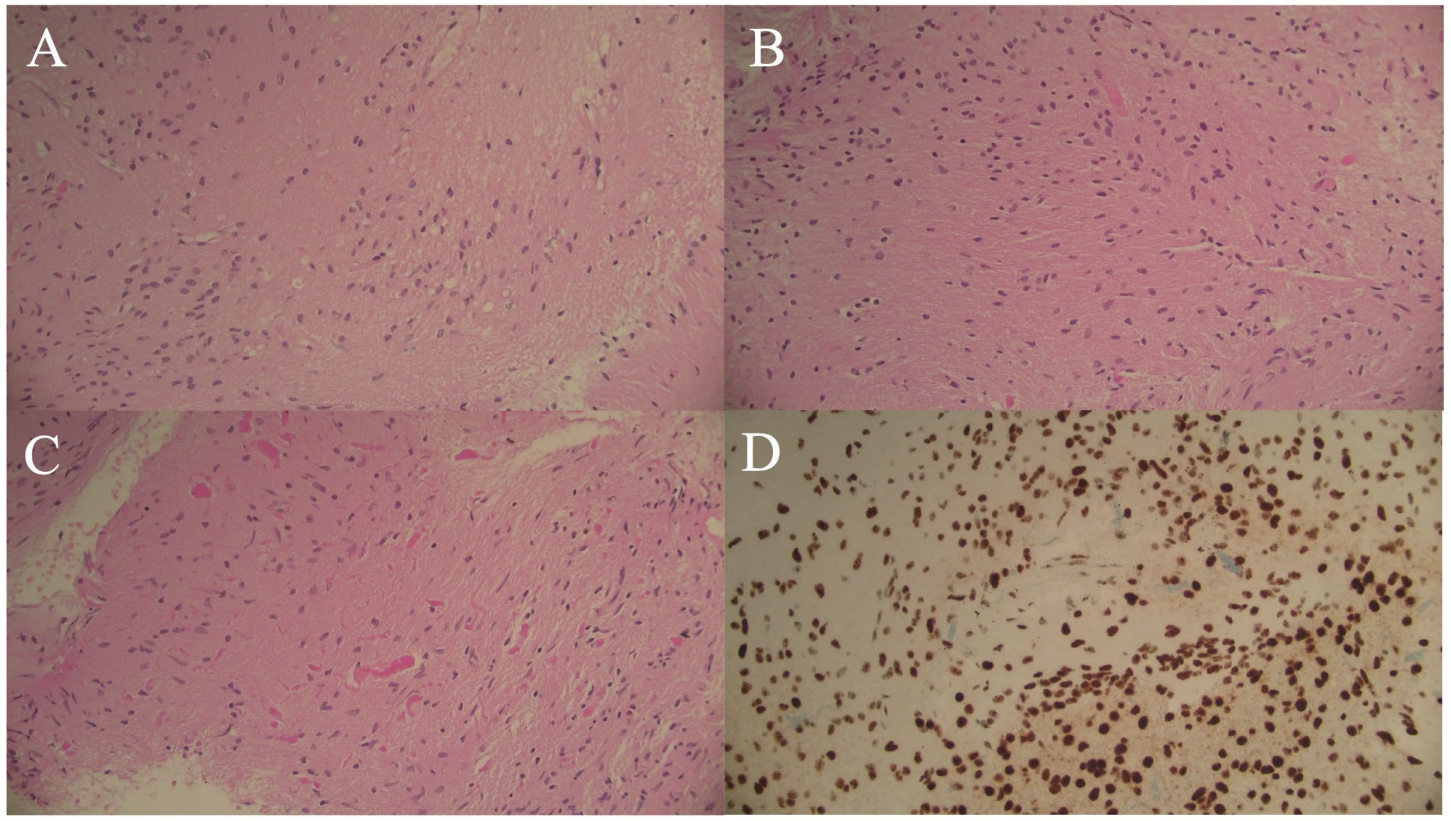
The histological appearance was that of a low-grade glioma with piloid features. The tumour was mostly of low cellularity with focal, poorly formed microcystic change **(A)** and cytologically bland **(B)**. In many areas numerous Rosenthal fibres were present **(C)**. ATRX expression was retained **(D)**. Scale = x200.

**Figure 3:**
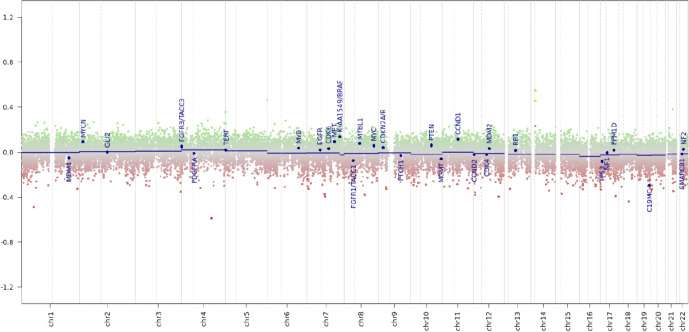
Copy number variation (CNV) plot based on DNA Illumina 850K EPIC lnfinium HD Methylation Array, depicting chromosome I to 22. Chromosomes are shown on the x-axis and logarithmic copy number ratio is shown on the y-axis. Gains or amplifications (green) are calculated as positive values and losses calculated as negative values. 29 brain tumour relevant genes are labelled according to chromosome location (blue).

Histology of the initial biopsy demonstrated a low-grade glial neoplasm with piloid features suggestive of pilocytic astrocytoma without mitotic activity, necrosis, or microvascular proliferation (**Figures 2A–C**). There were no oligodendroglial features. Immunohistochemistry for IDH1 was negative, p53 showed wild-type labelling, and there was retained expression of ATRX (**Figure 2D**). Single gene pyrosequencing (sequence analysis assay) for *IDH1, IDH2* and *H3-3A* was attempted due to insufficient DNA quantity for next-generation sequencing. Notably, a likely oncogenic *H3-3A* (NM_002107) p.G35W (c.103G>T) variant was detected. This is clinically referred to as *H3-3A* p.G34W. The sample was also confirmed to be *IDH*-wildtype, and no IDH1/2 variants were identified. Unfortunately, due to the limited sample size, there was insufficient tissue for further genomic sequencing, including allele quantification and BRAF testing. RNA based fusion and duplication screening was not performed due to insufficient sample. On a subsequent biopsy, DNA methylation array profiling was performed, which demonstrated a match to the methylation class family, “Pilocytic astrocytoma”, and methylation subclass classification, “posterior fossa pilocytic astrocytoma”, with calibrated scores of 0.94 and 0.93, respectively (Illumina 850K EPIC Infinium HD Methylation Array, DKFZ Molecular Neuropathology 2.0 classifier v11b4). Copy number variation (CNV) did not demonstrate any significant gains/amplifications or loss of genes (**Figure 3**). Subsequent molecular testing of genes *AKT1, AR, BRAF, EGFR, ERBB2, ESR, FOXL2, GNA11, GNAQ, IDH1, IDH2, KIT, KRAS, MAP2K1, MET, MYD88, NRAS, PDGFRA, PIK3CA and PTEN* were performed without any clinically significant variants detected. At this stage, a multidisciplinary consensus has been considered for three monthly MRIs for radiographic surveillance with 2mm growth observed on T2 weighted images 18 months post-diagnosis with no therapy thus far.

## Discussion and conclusions

This case study reports a rare *H3-3A* p.G35W(G34W) variant in an adolescent tectal plate glioma patient. The incidence of H3 alterations remains predominantly conserved to brain and bone tumours, with the *H3-3A* p.G35W(G34W) occurring almost exclusively in GCTB with isolated reports in spinal astrocytomas (21–23). Sloan et al. have previously reported a case of a seven-year-old boy diagnosed with a diffuse midline glioma in the thoracic spinal cord harbouring both H3 p.K27M and p.G34W missense mutations with co-occurring pathogenic mutations involving *PPM1D, PTPN11, FGFR1 and ATRX* (21, 22). Approximately 92% of GCTB and 17% of high-grade astrocytomas harbour a H3.3 glycine missense mutation (24). Given the paucity of data on this H3.3 p.G35W(G34W) mutation in paediatric glioma, methylation profiling was included in the patient workup to understand the projected natural history of the disease (25). Recently, DNA-methylation-based tumour classification has been demonstrated to significantly impact diagnosis and risk stratification, resulting in a changed diagnosis in 12% (129/1,104 cases) of patients with CNS tumours (25). Multiomic diagnostics, including methylation profiling, has now prospectively been shown to aid in the diagnosis and prognostication of gliomas (26) and is now available as a NATA (CLIA equivalent) accredited test in Australia (27). Given the stark differences in treatment and outcome, the diagnosis of PA by methylation profiling was reassuring and contributed to the expectant management of this patient.

Epigenetic regulation in paediatric gliomas is tightly regulated by H3 K27 and H3 K36 residues. Methylation of these residues is catalysed by methyltransferases, SETD2, NSD1, NSD2, and ASH1L through the addition of mono-, di- or trimethylation (28, 29). The proposed mechanism in which the missense mutation p.G35W(G34W) in GCTB drives oncogenesis is through complex interactions with epigenetic regulators and polycomb-group proteins (PcG) (30). PcGs form the polycomb repressive complex 2 (PRC2), which functions at the chromatin level alongside critical histone H3 lysine residues to maintain transcriptional silencing and regulation (31). Imbalance in this process has been proven to play a core role in human cancers (32).

H3.3 p.G35W(G34W) mutations studied in GCTB resulted in decreased H3 K36 methylation and increased H3 K27 methylation via steric hindrance of SETD2 catalytic activity (24). Similarly, H3.3 p.G35R/V(G34R/V) missense mutations in paediatric HGG affect trimethylation at the H3 K36 residue through abrogation of SETD2 binding with promotion of PRC2 activity and, subsequently, tumourigenesis (24, 33–35). Interestingly, in a study of 95 H3.3 p.G35R/V(G34R/V)-mutant gliomas 50% harboured *PDGFRA* mutations, where the p.G35R/V(G34R/V) missense is non-essential to tumour maintenance, but mutant *PDGFRA* spatial proximity to regulatory *GSX2* elements expressed in interneuron progenitors, resulting in downstream MAPK/ERK signalling (9). Chen et al. demonstrate that activating *PDGFRA* mutations provide strong selective pressures in driving gliogenesis and may promote an abnormal astrocyte-like state (9). On subsequent molecular testing, our patient did not harbour a mutant *PDGFRA*, but whether this patient with an H3.3 p.G35W(G34W) mutation arises from GSX2/DLX-expressing interneuron progenitors is unknown.

DNA methylation profiling has advanced the field of CNS tumour classification with its utility in aiding diagnostic accuracy by further delineating tumour subtypes, allowing for refined patient care and risk stratification (36). Methylation classifiers are only able to categorise tumours into known entities, which may misclassify rare tumours. As tectal gliomas are not standard subgroups, this case has been classified to the nearest known entity, which is PA. While existing methylation markers such as *MGMT* (O^7^-methylguanine-DNA methyltransferase) promoter hypermethylation have been utilised as a prognostic marker and predictor for chemotherapy sensitivity (37, 38), further cataloguing of methylation signature tumour classification remains an area of current focus. This classification has become necessary alongside clinical, histological and molecular features as defined in the fifth edition of the WHO CNS classification (3). Recently, diffuse hemispheric gliomas, H3 G34-mutant (DHG H3 G34m), have been recognised as a new glioma type within the current WHO CNS classification (23). However, only H3 G35R/V(G34R/V) are listed in association with DHG H3 G34m thus far, whilst G35W/L(G34W/L) variants are typically thought to be confined to GCTB (23).

While there is comparative evidence in which the mechanism of mutant H3.3 may contribute to tumourigenesis (31, 39), it is unclear whether these mechanics are relevant to our patient harbouring an H3.3 p.G35W(G34W) alteration without further investigation. Reassuringly, methylation profiling classified this case as posterior fossa pilocytic astrocytoma rather than a DHG H3 G34m. In this case, other genes known to be commonly mutated in DHG H3 G34m, including *TP53* and *ATRX*, were wildtype. Reliable molecular testing is required for tailored treatment, with challenges posed by limited tissue specimens and prioritising essential investigations (40). Circulating DNA (Ct-DNA), cell-free DNA (cfDNA), circulating RNAs (ctRNA) and microRNAs (miRNAs) detected in CSF is a promising diagnostic method of ‘liquid’ biopsy currently being clinically validated in paediatric CNS tumours (41, 42). The clinical utility of ‘liquid’ biopsy in this patient could be utilised for longitudinal genomic evaluation or reveal mutations that may not be detected in direct tissue samples due to tumour heterogeneity (43).

The patient highlighted in this case report remains clinically quiescent; however, due to the surgical nuance of tectal plate glioma resection, ongoing close monitoring via MRI is being performed to ensure best treatment practice. Frontline chemotherapy and small molecule pathway inhibitors could be considered for this patient. Dabrafenib and Trametinib have been proven efficacious in treating *BRAF* V600-mutant LGG, targeting MEK/ERK cell signalling (44). Trametinib, as a single agent in refractory and progressive LGG with MAPK/ERK signalling, has demonstrated significant responses in LGG cohorts, including non-*BRAF* mutated patients (45). Therefore, if this tumour was to transform clinically, further testing would be required to re-evaluate the MAPK pathway to determine if targeted therapy is a potential avenue of treatment in the case of this BRAF wildtype patient. Radiotherapy is typically avoided in paediatric, adolescent, and young adult low-grade glioma currently (19, 20). However, proton therapy may hold a more promising treatment avenue if there is a recrudescence of the patient’s symptoms. While limited in availability, proton therapy is quickly becoming a superior treatment strategy for low-grade gliomas, with far less off-target effects and excellent survival rates (46).

Here, we have demonstrated, for the first time, a novel *H3-3A* p.G35W(g34W) variant in an adolescent case of low-grade tectal plate glioma. Typically, only seen in GCTB, *H3-3A* p.G35W(G34W) alteration has not been reported in the supratentorial setting, with prior documented cases limited to diffuse spinal cord astrocytomas (22). While biopsies of low-grade gliomas are not typically performed in adult patients, it is possible that G34-mutant gliomas are more prevalent than currently reported, and further methylation would provide a supportive diagnostic classification where genomic profile may be ambiguous. While low-grade tectal plate glioma is typically managed without treatment, DHG H3, require surgical intervention and have a poor prognosis. Therefore, our case demonstrates that methylation profiling is critical to help determine CNS tumour classification and ensure accurate diagnosis and prognostication, tailored treatment, and monitoring for potential transformation.

## Data Availability

The original contributions presented in the study are included in the article/**Supplementary Material**. Further inquiries can be directed to the corresponding author.

## Glossary

PA: Pilocytic astrocytoma
DA: Diffuse astrocytoma
WHO: World Health Organization
LGG: Low-grade glioma
MAP: Mitogen-activated protein
*BRAF*: Proto-oncogene encoding protein, B-raf
HGG: High-grade gliomas
DMG: Diffuse midline glioma
OPC: Oligodendroglial precursor cell
DHG: Diffuse hemispheric glioma
H3-3A p. G35W (G34W): Amino acid change of glycine to tryptophan at the 35^th^ amino acid in H3 histone proteins
H3 K27M: Amino acid change of lysine to methionine at the 28th amino acid (p.K28M) in H3 histone proteins
H2A: H2B, H3, Canonical histone protein subunits (H3.1 and H3.2), H4
H3 (H3.3): Non-canonical histone protein subunits
GCTB: Giant cell tumour of bone
DNA: Deoxyribonucleic acid
CSF: Cerebral spinal fluid
OS: Overall survival
MRI: Magnetic resonance imaging
*IDH1/2*: Isocitrate dehydrogenase 1 & 2
*ATRX*: Gene encoding Transcriptional regulator ATP-dependent helicase
*AR*: Gene encoding androgen receptor
*EGFR*: Gene encoding epidermal growth factor receptor
*ERBB2*: Gene encoding ErbB2 tyrosine kinase of epidermal growth factor receptor (also known as HER2)
*ESR*: Gene encoding oestrogen receptor
*FOXL2*: Gene encoding transcription factor forkhead box protein L2
*GNA11*: Gene encoding gonadotropin-releasing hormone receptor
*GNAQ*: Gene encoding protein guanine nucleotide-binding protein
*KIT*: Proto-oncogene encoding a receptor tyrosine kinase
*KRAS*: Gene encoding protein K-ras
*MAP2K1*: Gene encoding protein MEK1 protein kinase
*MET*: Proto-oncogene encoding hepatocyte growth factor receptor
*MYD88*: Gene encoding cytosolic adapter protein
*NRAS*: Gene encoding N-Ras protein
*PDGFRA*: Gene encoding platelet-derived growth factor receptor alpha
*PIK3CA*: Gene encoding p110 alpha protein
*PTEN*: Gene encoding phosphatase and tensin homolog
*PPM1D*: Gene encoding protein phosphatase, Mg2+/Mn2+ dependent 1D
*PTPN11*: Gene encoding tyrosine phosphatase SHP-2
SETD2: SET domain containing 2, histone lysine methyltransferase
*GSX2*: Gene encoding GS homeobox 2 protein
*TP53*: Gene encoding tumour suppressing TP53 protein
*MGMT*: Gene encoding O⁶-methylguanine-DNA methyltransferase
*DLX*: Gene encoding homeobox transcription factor
NSD1/2: Nuclear receptor-binding SET domain of H3 Lysine 36 methyltransferases
ASH1L: Histone-lysine N-methyltransferase
PRC2: Polycomb repressive complex 2
CNS: Central nervous system
NATA: National Association of Testing Authorities
CLIA: Clinical Laboratory Improvement Amendments
Ct-DNA: Circulating DNA
CfDNA: Cell-free DNA
CtRNA: Circulating RNAs
MiRNAs: MicroRNAs
