# Supplementary figures and images for: Case Report: Identification of rare H3-3A p.G35W variant in a case of adolescent tectal plate low-grade glioma
Source: Front Oncol. 2026 Apr 30;16:1772874. doi: 10.3389/fonc.2026.1772874 (PMC13171345; doi:10.3389/fonc.2026.1772874)

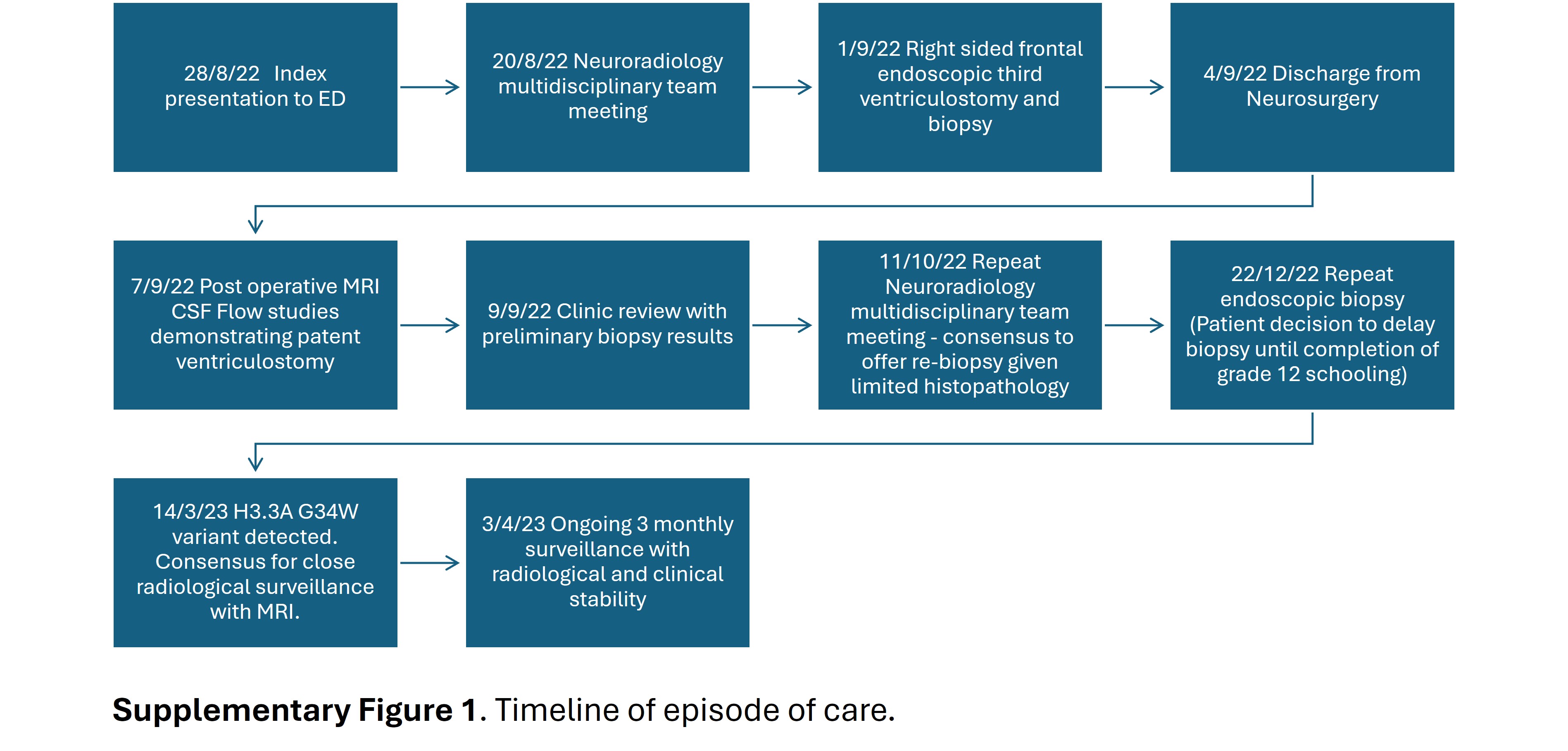

Supplement: Supplementary file 1 [file Image1.jpg]
